# The neural effects of acupuncture for stroke: a protocol for systematic review and meta-analysis based on fMRI

**DOI:** 10.3389/fnins.2024.1443834

**Published:** 2024-09-06

**Authors:** Shaojie Cai, Yihao Zhou, Siyu Yang, Yichang Liu, Yixiao Han, Dongyan Wang, Shuai Shi

**Affiliations:** ^1^Heilongjiang University of Chinese Medicine, Harbin, China; ^2^The Second Affiliated Hospital, Heilongjiang University of Chinese Medicine, Harbin, China

**Keywords:** stroke, acupuncture, neural effects, systematic review, meta-analysis, protocol

## Abstract

**Background:**

Stroke is one of the most common causes of death and is the main cause of persistent and acquired disability in adults worldwide. Acupuncture is recommended by the World Health Organization as an alternative and complementary strategy for stroke treatment. However, the mechanism of the neural effects of acupuncture on stroke is still lacking a uniform conclusion. The purpose of this study is to clarify the neural effects of acupuncture for stroke from the perspective of neuroimaging.

**Methods:**

Seven databases, including PubMed, Embase, Cochrane Library, Chinese National Knowledge Infrastructure, VIP Database, Wan-Fang Data, and Chinese Biomedical Database, will be searched comprehensively according to the search strategy. All of them will be retrieved from the date of database establishment to December 31, 2023. All randomized controlled trials and observational studies will be considered for inclusion. The risk of bias will be assessed by the Cochrane Risk of Bias tool. Functional imaging of the whole brain in the resting functional state will be the primary outcome. A meta-analysis of primary outcome will be performed using Seed-based D Mapping with Permutation of Subject Images software. The data will be synthesized using a random effects model. Meta-analysis of other clinical data will be performed using RevMan 5.3 software. Publication bias will be assessed through funnel plots. Reports will adhere to the Preferred Reporting Items for Systematic Review and Meta-Analysis.

**Conclusion:**

This study will aggregate the results of independent studies to provide overall evidence for the neural effects of acupuncture for stroke.

**Clinical Trial Registration:**

https://www.crd.york.ac.uk/PROSPERO/display_record.php?RecordID=500306, Identifier CRD42024500306.

## Introduction

Stroke is defined as an acute focal injury of the central nervous system arising from a vascular cause such as cerebral infarction, or intracerebral hemorrhage (Sacco et al., 2013). It is one of the most common causes of death and is the main cause of persistent and acquired disability in adults world. According to the Global Burden of Disease Study 2019, there were 12.2 million incident cases of stroke in the worldwide (GBD 2019 Stroke Collaborators, 2021). With an aging population and an increasing prevalence of unhealthy lifestyles, the disease burden of stroke in China is on an explosive growth trend (Tu et al., 2023). Motor dysfunction is the main clinical manifestation, additionally, survivors may also suffer from cognitive dysfunction, swallowing disorders, aphasia, paresthesia and other symptoms. It has been estimated that more than 90% of the burden of stroke can be attributed to modifiable factors (Feigin et al., 2016). Interventions that target modifiable factors can improve clinical symptoms and thus reduce the adverse impact of disease burden (Feigin et al., 2014).

Acupuncture, which involves inserting thin needles into the skin or deep tissues of the body, is one of the distinctive techniques of traditional Chinese medicine and is recommended by the World Health Organization as an alternative and complementary strategy for stroke treatment (Li and Wang, 2013; Chavez et al., 2017). A narrative review indicated that acupuncture had a positive effect on motor recovery, dysphagia, shoulder pain, cognitive impairment, depression and other symptoms in stroke patients (Birch and Robinson, 2022). Evidence showed that the mechanisms of acupuncture in the treatment of stroke mainly include promoting neurogenesis and cell proliferation, regulating cerebral blood flow in ischemic areas, anti-inflammation and anti-apoptosis (Chavez et al., 2017).

Functional magnetic resonance imaging (fMRI) is a non-invasive imaging technique with high temporal and spatial resolution that measures neuronal activity by monitoring hemodynamic responses (Glover, 2011). A growing number of acupuncture studies have begun to apply fMRI techniques to explore neural effects associated with therapeutic responses to better understand the underlying mechanisms of acupuncture (Zhang et al., 2021a). In recent years, researchers have studied the central nervous effect of acupuncture in the treatment of stroke based on fMRI, and found that acupuncture can activate functional activities in specific brain regions, such as motion-related networks and language-related brain areas (Zhang et al., 2021b). However, the results of various studies are not completely consistent, and the mechanism of the neural effects of acupuncture on stroke is still lacking a uniform conclusion.

At present, there have been systematic reviews or meta-analyses to comprehensively analyze the fMRI data of acupuncture for stroke (Lv et al., 2021; Zhang et al., 2021b). Unfortunately, these studies only looked at post-stroke motor dysfunction and did not discuss other symptoms. It is well known that the symptoms after a stroke are varied.

Here, we hope to clarify which brain area plays a key role in the effect of acupuncture by conducting a quantitative meta-analysis of published imaging studies of acupuncture for stroke. The results of this study will determine whether acupuncture has a different effect on brain activity in stroke patients compared to non-acupuncture treatment, and will also further analyze whether different clinical manifestations of stroke cause different brain regions to be activated. The results of this study may provide new insights into the neural effects mechanism of acupuncture treatment for stroke in the future.

## Methods

### Study registration

This protocol has been registered in the PROSPERO, and the registration number is CRD42024500306. All reporting procedures have been implemented in strict accordance with the Preferred Reporting Items for Systematic Review and Meta-Analysis Protocols 2015 (PRISMA-P) (Shamseer et al., 2015).

### Literature search

We plan to search the following databases: PubMed, Embase, Cochrane Library, Chinese National Knowledge Infrastructure (CNKI), VIP Database, Wan-fang Data, and Chinese Biomedical Database (CBM). All of them will be retrieved from the date of database establishment to December 31, 2023. The search mode used will be a combination of medical subject words and free words. Publication language of all literature will be limited to English and Chinese. The search strategy takes PubMed as an example, and shown in Table 1.

**Table 1:** The search strategy for PubMed.

Order	Strategy
#1	Search: “Stroke”[Mesh]
#2	Search: (apoplexy[Title/Abstract]) OR (cerebral infarction[Title/Abstract]) OR (cerebral stroke[Title/Abstract]) OR (cerebral hemorrhage[Title/Abstract]) OR (hemorrhagic apoplexy[Title/Abstract]) OR (cerebrovascular accident[Title/Abstract]) OR (brain vascular accident[Title/Abstract])
#3	#1 OR #2
#4	Search: “Acupuncture”[Mesh]
#5	Search: (needle[Title/Abstract]) OR (electroacupuncture[Title/Abstract]) OR (electrical acupuncture[Title/Abstract]) OR (scalp acupuncture[Title/Abstract]) OR (manual acupuncture[Title/Abstract]) OR (ear acupuncture[Title/Abstract])
#6	#4 OR #5
#7	Search: “magnetic resonance imaging”[Mesh]
#8	Search: (magnetic resonance image) OR (NMR imaging) OR (neuroimaging) OR (MRI scans) OR (functional MRI) OR (fMRI) OR (spin echo imaging)
#9	#7 OR #8
#10	Search: (humans) NOT (animals)
#11	#3 AND #6 AND #9 AND #10

### Inclusion and exclusion criteria

#### Participants

The participants of the experimental group must be stroke patients, who can suffer from motor dysfunction, cognitive dysfunction, dysphagia and other complications. The control group would allowed to recruit healthy subjects as study participants, regardless of gender, age, occupation, education level, etc.

#### Intervention

The experimental group can use acupuncture therapies, and the mode may be body acupuncture, scalp acupuncture, electrical acupuncture or ear acupuncture. There are no additional restrictions on the prescription, course or frequency of acupuncture.

#### Comparison

The control group can be treated with any non-acupuncture therapy, allowing patients to receive basic treatment in accordance with the treatment guidelines, but should be consistent with the treatment measures of the experimental group (e.g., secondary prevention, rehabilitation other than acupuncture).

#### Outcomes

(1) *Primary outcome.* The results include functional imaging of the whole brain in the resting functional state. Peak coordinates (x, y, z) and effect sizes (*t*-value, or *z*-value or *p*-value) reported by the standard stereotactic space Talairach or Montreal Neurological Institute (MNI). (2) *Secondary outcome.* Outcome measures reflecting clinical efficacy and safety, such as the National Institutes of Health Stroke Scale (NIHSS), the modified Rankin Scale (mRS), the Barthel Index (BI).

#### Study type

All randomized controlled trials (RCTs) and observational studies published on the subject will be considered for inclusion. Reviews, animal experiments, case reports, case series, study protocols and other non-RCT studies will be excluded.

### Study selection

Literature selection will be conducted independently by two reviewers (YZ and SY). All literature that meet the inclusion criteria will be managed using Endnote (version X9) software. After removing duplicates, they will screen the titles, abstracts, and the further reading the full text of the remaining records to identify eligible studies. Any disagreements will be resolved through team discussion or negotiation. Reasons for exclusions will be recorded in detail. Details of the selection process will be presented in the results of the study by a PRISMA flowchart.

### Data extraction

Two data managers (YL and YH) will independently extract data according to a unified data extraction table developed in advance. If data is insufficient or missing, the corresponding author will be contacted by email for further information. If we do not receive a response, we will exclude the study and indicate the reason in the flow chart. The data extraction table mainly include: basic information, trial characteristics, research methods, intervention and control measures, imaging information (scan parameters, algorithm, coordinates, *t*-value, or *z*-value or *p*-value), secondary outcomes of the study. Outcome data on treatment effects will be collected from the immediate assessment after the intervention or the assessment closest to the end of therapy. Our study does not consider follow-up results. The data needs to be cross-checked after extraction, and any disagreements will be resolved through team discussion or negotiation.

### Quality assessment

Based on the assessment methods used in previous imaging meta-analyses (Iwabuchi et al., 2015; Zhao et al., 2022), we will employ a modified version of the checklist to assess the methodological quality of fMRI studies. The checklist include two categories with 13 items, mainly focusing on sample size, methodology and reporting of results. The total score is 20 points. The higher the score, the better the methodological quality. Additionally, we will use the Cochrane Bias Risk Tool (Higgins et al., 2011) to assess the overall quality of included studies across six domains: random sequence generation, allocation concealment, blinding, incomplete outcome data, selective reporting, and other sources of bias. The two reviewers (YZ and SY) will complete the quality assessment independently, and any disagreements will be resolved through team discussion or negotiation.

### Heterogeneity assessment

Due to the broad scope of our study, the heterogeneity of fMRI studies will pose a challenge to our research. In particular, different clinical manifestations after stroke may show that different brain regions are activated, so there may be a higher risk of heterogeneity. Heterogeneity assessment will be performed by extracting MNI peak coordinates and quantifying with *I^2^*. *I^2^* < 50% indicates low heterogeneity. Additionally, the acupuncture procedures may also lead to heterogeneity. We will evaluate acupuncture clinical trial methods by Revised Standards for Reporting Interventions in Clinical Trials of Acupuncture (STRICTA) (MacPherson et al., 2010).

### Data synthesis

A meta-analysis of primary outcomes will be performed using Seed-based D Mapping with Permutation of Subject Images software (version 6.21).^1^ Data analysis shall be carried out in MNI spatial coordinates. If the research results are not reported in MNI spatial coordinates, the coordinates will be uniformly converted to MNI types firstly. The mean analysis, heterogeneity test, sensitivity analysis, and meta-regression analysis will then be continued using appropriate software packages. Finally, the results will be presented in an Excel spreadsheet, the 3D coordinate data will be mapped to the MNI template with MRIcron software.^2^ The statistical analysis threshold of this voxel meta-analysis is *p* < 0.05 and voxel extent ≥10.

Meta-analysis of other clinical data will be performed using RevMan5.3 software. In general, the results of stroke scales, such as NIHSS, mRS and BI, belong to continuous variable data. Continuous variables will be expressed as standardized mean difference with confidence interval of 95%. And other categorical variables will be represented by risk ratio. The result will be presented as a forest map and the funnel plot will be used to assess publication bias.

### Subgroup analysis

If the heterogeneity between studies is significant, we will perform subgroup analysis based on different complications. Other factors that may affect the stability of the results may also be reasons for further subgroup analysis.

### Grading the quality of evidence

We will evaluate the quality of evidence according to the Grading of Recommendations Assessment, Development and Evaluation (GRADE) (Balshem et al., 2011). The quality grade may be rated down for the following reasons: risk of bias, inconsistency, indirectness, imprecision, publication bias. It is divided into four grades: high, moderate, low, very low.

## Discussion

Stroke has become a major chronic noncommunicable disease that endangers the health of the Chinese people, bringing a serious burden to families and society (Wang et al., 2017). Stroke survivors may have multiple complications at the same time, and the mechanisms of acupuncture is multi-faceted and multi-channel (Wen et al., 2021). Therefore, our systematic review and meta-analysis will include all fMRI studies on acupuncture for stroke, hoping to explore the neural effects from a holistic perspective and further explore the similarities and differences between the overall effects and subgroup results through subgroup analysis. Our findings will help elucidate the neurological effects of acupuncture in stroke treatment, reveal the brain regions that play a key role, and provide new ideas for acupuncture treatment of stroke.

Meta-analysis based on fMRI research is a method of brain region localization analysis based on voxel coordinates, which realizes brain region localization through three-dimensional Gaussian smoothing and permutation tests of relevant coordinates included in the study (Zhao et al., 2022). However, meta-analyses based on fMRI studies are rarely reported, which makes our report on protocol necessary and extremely valuable. On the one hand, peer review can examine the correctness of our methodology; On the other hand, meta-analysis following published protocol can reduce bias and improve the quality of research results. In conclusion, our study can aggregate the results of independent studies to provide overall evidence for the neural effects of acupuncture for stroke, and can provide some valuable references for future research on the neural effects of acupuncture for stroke, such as the selection of seed points or interest brain areas.

### Strengths and limitations of this study

- This study can aggregate the results of independent studies to provide overall evidence for the neural effects of acupuncture for stroke.
- We will conduct our research in strict accordance with the methodological guidelines of systematic review and meta-analysis.
- Based on the assessment methods used in previous imaging meta-analyses, we will employ a modified version of the checklist to assess the methodological quality of fMRI studies. Additionally, the Cochrane Bias Risk Tool will be used.
- A meta-analysis may not be possible for certain outcomes due to a limited number of eligible studies and due to heterogeneity of the studies.

## Footnotes

^1^https://www.sdmproject.com/

^2^https://www.nitrc.org/projects/mricron
